# The frequency of the known mitochondrial variants associated with drug-induced toxicity in a Korean population

**DOI:** 10.1186/s12920-021-01153-0

**Published:** 2022-01-03

**Authors:** Vinh Hoa Pham, Van Lam Nguyen, Hye-Eun Jung, Yong-Soon Cho, Jae-Gook Shin

**Affiliations:** 1 Department of Pharmacology and Pharmacogenomics Research Center, Inje University, College of Medicine, 633-165 Gaegum-Dong, Jin-Gu, Busan, Republic of Korea; 2 Department of Precision Medicine, SPMED Co., Ltd., Busan, 46508 Republic of Korea; 3 Department of Pharmacology and Clinical Pharmacology, PharmacoGenomics Research Center, Inje University College of Medicine, Busan, 47392 Republic of Korea; 4 Center for Personalized Precision Medicine of Tuberculosis, Inje University College of Medicine, Busan, Republic of Korea

**Keywords:** Mitochondria, Polymorphism, Drug-induced toxicity, Korean population

## Abstract

**Background:**

Few studies have annotated the whole mitochondrial DNA (mtDNA) genome associated with drug responses in Asian populations. This study aimed to characterize mtDNA genetic profiles, especially the distribution and frequency of well-known genetic biomarkers associated with diseases and drug-induced toxicity in a Korean population.

**Method:**

Whole mitochondrial genome was sequenced for 118 Korean subjects by using a next-generation sequencing approach. The bioinformatic pipeline was constructed for variant calling, haplogroup classification and annotation of mitochondrial mutation.

**Results:**

A total of 681 variants was identified among all subjects. The *MT-TRNP* gene and displacement loop showed the highest numbers of variants (113 and 74 variants, respectively). The m.16189T > C allele, which is known to reduce the mtDNA copy number in human cells was detected in 25.4% of subjects. The variants (m.2706A > G, m.3010A > G, and m.1095T > C), which are associated with drug-induced toxicity, were observed with the frequency of 99.15%, 30.51%, and 0.08%, respectively. The m.2150T > A, a genotype associated with highly disruptive effects on mitochondrial ribosomes, was identified in five subjects. The D and M groups were the most dominant groups with the frequency of 34.74% and 16.1%, respectively.

**Conclusions:**

Our finding was consistent with Korean Genome Project and well reflected the unique profile of mitochondrial haplogroup distribution. It was the first study to annotate the whole mitochondrial genome with drug-induced toxicity to predict the ADRs event in clinical implementation for Korean subjects. This approach could be extended for further study for validation of the potential ethnic-specific mitochondrial genetic biomarkers in the Korean population.

**Supplementary Information:**

The online version contains supplementary material available at 10.1186/s12920-021-01153-0.

## Background

Mitochondria are organelles that play a central role in cellular energy suppliers. They have their own genome and genetic code, and an exceptionally high mutation rate [1]. At least 100 mutations are expected to be observed in almost 90% of non-proliferating cells, while no other cell types have fewer than 10 mutations by the age of 70 years [2]. Polymorphism of mitochondrial DNA (mtDNA) is associated with various pathophysiology, and could explain diverse vulnerability to the diseases or drug toxicity [3]. The high variability of human mitochondria has been studied in the context of common diseases. Ninety-five mitochondrial markers in the Mitomap database have been confirmed to be pathogenic as of October 2021 [4]. Pathogenic mtDNA mutations were reported to be common in the general population, and are also present in some major haplogroups. Therefore, many healthy individuals carry potential harmful variants. At least 1 in every 200 healthy subjects harbours a pathogenic variant that can be a potential cause of disease in the next generation [5].

In addition, mitochondrial polymorphisms were reported to be associated with drug responses, and can reveal the role of mtDNA variation in susceptibility to drug toxicity. It has been reported that variations in mtDNA can result in differences in mitochondrial function that, in turn, may lead to idiosyncratic drug-induced toxicity [6]. Limited information, however, is available about the roles of mitochondrial haplogroups in susceptibility to drug-induced toxicity. Anti-retroviral therapy (ART), antibiotics and chemotherapeutic agents are well known drug classes in relation to the mitochondria mediated drug toxicities [7]. The mtDNA mutations and copy numbers have been proposed as potential biomarkers to monitor therapeutic responses and prognosis in cancer treatments, although the mechanisms are not well established [8, 9]. There are reports that specific mtDNA haplogroups are associated with ART-induced peripheral neuropathy and metabolic disorders from clinical studies conducted in European patients [10–15]. The susceptibility of antibiotics toxicity is also well known to associated with mtDNA polymorphisms, especially in mitochondrial 12S rRNA and 16S rRNA genes which play an important role in preventing or inducing toxicity [16].

The mtDNA has a unique genetic feature known as ‘heteroplasmy’, which allows mutant and wild-type mtDNAs to coexist. It is well known that healthy individuals harbour relatively low levels (< 1%) of mtDNA heteroplasmy in general. It is known that the mtDNA heteroplasmy increases throughout the lifespan, and the initial mutant mtDNA will be the predominant type within a specific cell. Once a certain threshold level of mutant mtDNA is above the normal range, the mutant load influences on the cellular and patient phenotypes. Hence, a low level of heteroplasmy would be linked to late-onset of related diseases in an individual subject [17]. Since the Next-generation sequencing (NGS) technology is a tool to identify and quantify the level of heteroplasmy at the 1% range, this high throughput technology has been implemented extensively towards decoding the mitochondrial genome in many ethnic populations [18–22]. Two reports are available so far for the NGS-based mitochondrial genome study in the Korean subjects [23, 24], and they found the similar profile of mitochondrial genetic variants to that in the Japanese and Chinese Han populations [19, 25]. However, those reports are not issued on the potential implementation of those mtDNA variants as potential biomarkers for the prediction of drug induced toxicity.

In this study, therefore, the mtDNA genetic profiles of 118 Korean subjects were analysed by using the NGS approach, in order to investigate the distributions and frequencies of known mtDNA variant biomarkers associated with diseases and drug-induced toxicity in a Korean population.

## Methods

### Sample collection and DNA extraction

DNA samples were harvested from peripheral blood that were selected from biobank in Pharmacogenomics Research Center, Inje University, Korea. In totally, 118 independent Korean subjects, all of whom provided written informed consent to participate in the genotyping analysis, were adults with a mean age of 37.1 years (range 19–65 years), and a mean BMI of 22.1 (range 15.1–34.1 kg m^−2^). The study population consisted of 65 males (55%) and 88 (74.6%) non-smokers. Sixty-eight subjects were collected from multicentre for Personalised Precision Medicine of Tuberculosis from 2018 to December 2020. The subjects with age from 18 to 65 years old, were diagnosed with drug-susceptible tuberculosis and treated with first-line anti-tuberculosis drugs (rifamycin, isoniazid, pyrazinamide and ethambutol). The subjects had no adverse drug reactions, no comorbidities and normal laboratory test results during treatment. The remaining 50 individuals were selected from 1000 Korean healthy subjects that were recruited from September 1, 2005 to August 31, 2015 with the support of Ministry of Health and Welfare, Korea for establishment of a pharmacogenetics database for the healthy Korean adults. The selected subjects were individuals over 19 years old without any recorded diseases at the time of sample collection. The characteristic of subjects in this study were described as in Additional file 1: Table S1.

Blood samples were collected at the time of recruitment and frozen at − 80 °C. Total DNA was isolated from peripheral blood samples using a QIAamp DNA Blood Mini Kit (Qiagen, German) according to the manufacturer’s instructions. DNA purity and concentration were measured using a NanoDrop 2000 Spectrophotometer (Thermo Fisher Scientific, USA).

### Amplification of mitochondrial long-range fragments

A well-established method for exclusive amplification of mitochondrial long-range fragments as described by Gould et al. [26] was used to avoid the co-amplification of nuclear genomic DNA. The PCR primers designed for the study included MT-L1_F (5ʹ-AAATCTTACCCCGCCTGTTT-3ʹ), MT-L1_R (5ʹ-AATTAGGCTGTGGGTGGTTG-3ʹ), MT-L2_F (5ʹ-GCCATACTAGTCTTTGCCGC- 3ʹ), and MT-L2_R (5ʹ-GGCAGGTCAATTTCACTGGT-3ʹ) to amplify entire sequence of a mitochondrial genome. We used an Applied Biosystems (GeneAmp PCR system 9700, Thermo Fisher Scientific, USA) for analysing long-range fragments 1 and 2 (MT-L1 and MT-L2, respectively) of mitochondrial genomes (~ 8.5 kb each), as described by McElhoe et al*.* The PCR was performed using 50-μl reaction mixtures containing 200 ng of genomic DNA with LA Taq® and 2 × GC buffer (TaKaRa, Japan) [27]. The PCR conditions for both the MT-L1 and MT-L2 fragments consisted of an initial denaturation step at 94 °C for 1 min followed by 30 cycles of denaturation at 94 °C for 30 s, annealing at 59 °C for 40 s, extension at 72 °C for 9 min, and a final extension step of 72 °C for 10 min. The DNA template (9947A) from the National Institute of Standards and Technology (NIST, USA) [28] was utilised as the mitochondrial standard DNA for sequencing.

### Mitochondrial whole-genome sequencing

DNA libraries with an expected insert size of ~ 200 bp were prepared using NEBNext (UK) in accordance with the instruction manual provided [29], and optimised for our samples. The concentrations of mitochondrial libraries were measured using the QuantiFluor® ONE dsDNA system (Promega, USA). Equimolar amounts of the 60 indexed libraries were pooled to obtain a 4-nM mixture. After denaturation and further dilution, the final 16 pM of this mixture was loaded into an Illumina cartridge. Sequencing was performed using the Illumina MiSeq Reagent kit v2 (300 cycles) and MiSeqDx instrument (Illumina, USA) in accordance with the manufacturer’s instructions.

### Analysis of mitochondrial genomes

**Fig. 1:**
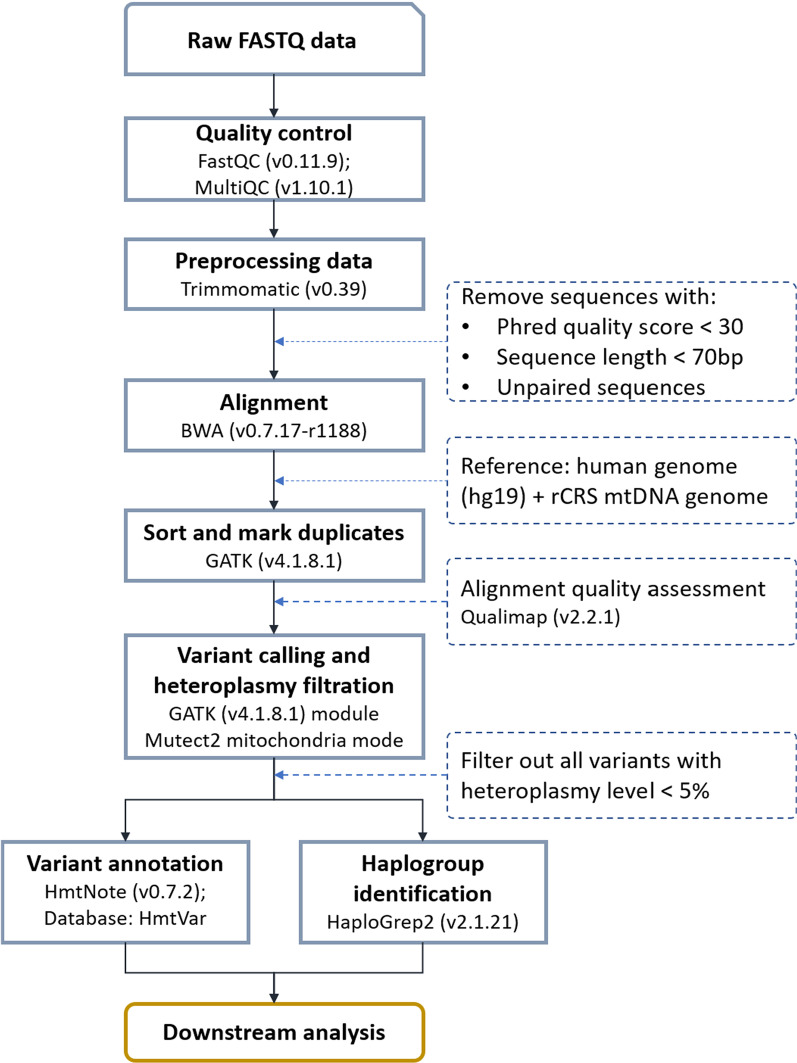
The flowchart of bioinformatics analysis pipeline in this study. rCRS: the revised Cambridge Reference Sequence; mtDNA: mitochondrial DNA

A bioinformatics pipeline was developed to reconstruct and analyse human mtDNA from high-throughput sequencing data as shown in Fig. 1. The sequencing data obtained from the Illumina MiSeqDx were quality-controlled using FastQC (version 0.11.9) [30] and MultiQC (version 1.10.1) [31]. All adapters and sequences with a quality score < 30 and sequence length < 70 bp were removed using Trimmomatic (version 0.39) [32]. In the alignment process, to minimise the nuclear mitochondrial DNA segment (NUMT), the qualified data of each sample were aligned simultaneously to human genome reference hg19 based on the revised Cambridge Reference Sequence (rCRS) of human mitochondrial DNA [33] using BWA (version 0.7.17-r1188) [34]. Regarding downstream alignment processes such as conversion from Sequence Alignment/Map (SAM) to Binary Alignment/Map (BAM) format and marking and removing duplicates were performed using GATK (version 4.1.8.1). The final alignment assessment was performed using Qualimap (version v.2.2.1) [35]. Variants and heteroplasmy were called using GATK Mutect2 (version 4.1.8.1) in mitochondrial mode. All variants were filtered for a heteroplasmy level < 5%. Other filtering processes were in accordance with the instructions of the GATK best practices for mitochondrial genomes [36]. The results of the variant-calling pipeline were confirmed based on the NIST mtDNA standard (GM09947A) [28] before applying to real samples. Finally, all variant-calling pipelines were combined. The variants were annotated using HmtNote (version 0.7.2) [37] against the HmtVar database [38]. The haplogroups and sub-haplogroups of all samples in the cohort were defined based on PhyloTree 17 nomenclature that is integrated in Haplogroup2 [39].

## Results

### Distribution of mitochondrial variants in the sequenced mtDNAs

The bioinformatics pipeline was confirmed to be consistent with the variant-calling pipeline for the mtDNA standard sequence [28]. According to NIST [40], the percentage of heteroplasmy for m.1393G > A was 17.4 ± 1.7% and that for m.7861T > C was 74.6 ± 14.5%, while the values in our study were 17.4 ± 0.6% and 89.5 ± 0.1%, respectively. These results confirmed the accuracy of the bioinformatics pipeline and experimental procedure for mtDNA sequencing and analysis. The average mtDNA coverage for the examined samples was 2601X ± 685X (detailed information of average coverage for each sample was shown in Additional file 1: Figure S1). The average read length for all paired sequences was 203 ± 15 bp.

**Fig. 2:**
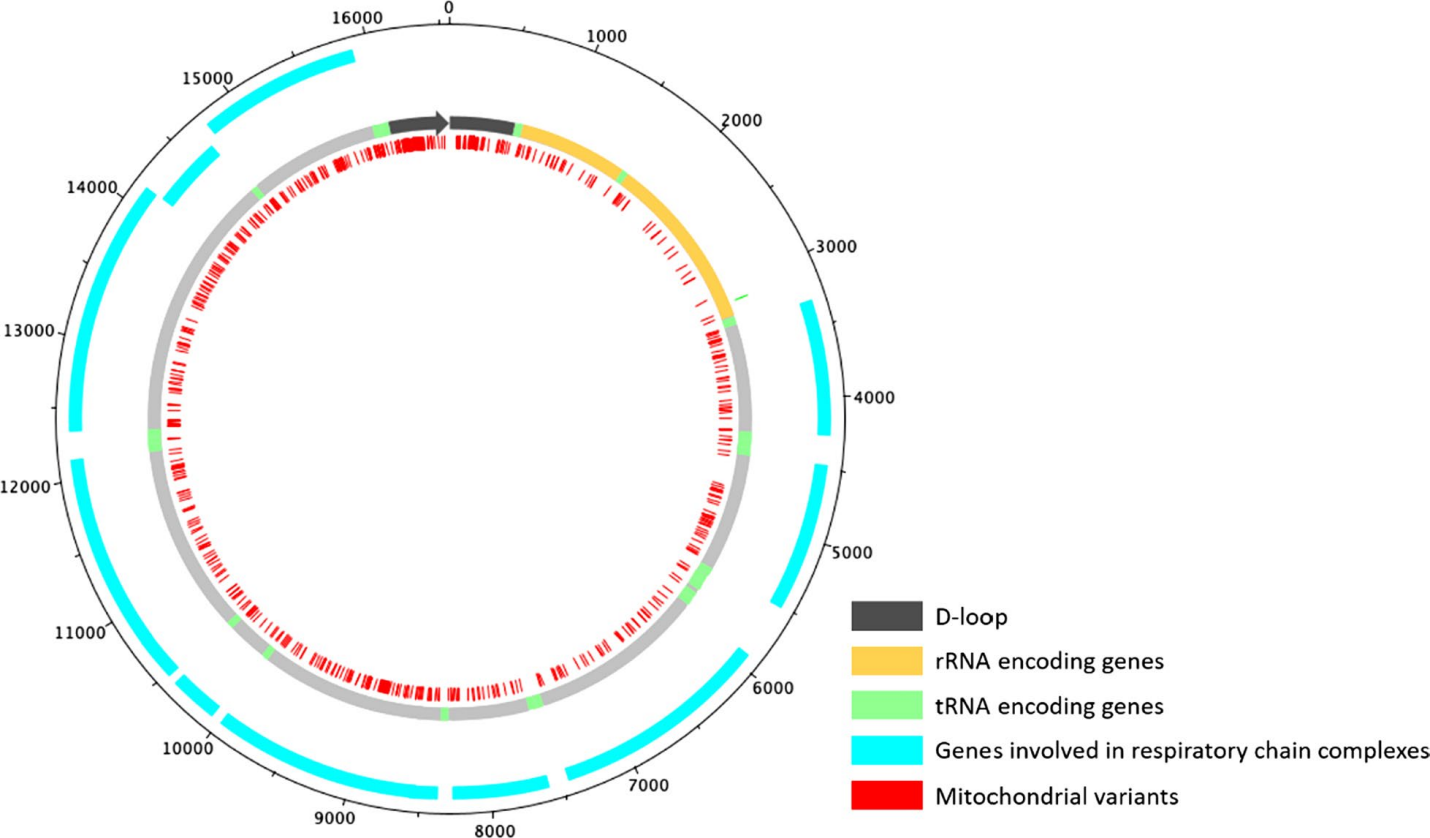
Distribution of variants in mitochondrial genomes in this study. Mitochondrial variants (red) in tRNA genes (green), rRNA genes (orange) and genes involved in mitochondrial respiratory chain complexes (blue), in the Korean population in the present study (*n* = 118) according to the Revised Cambridge Reference Sequence (NC_012920.1). The mitochondrial genome was generated by using DNAPlotter [42]

We identified a total of 681 variants called from 118 individuals (Fig. 2). Most of the variable positions were transitions (86.5% overall), while the overall percentage of transversions was about 3%. Our study showed that transitions were dominant, as reported previously for human mitochondrial genomes [41]. Indel polymorphism was detected at a rate of 8.5% among all individuals. The median of the mtDNA variants was 38 (range 26–55). No variants were found in 9/22 tRNA-coding genes. *MT-TRNP* gene and the displacement loop (D-loop) had the most polymorphisms (113 and 74 variants, respectively) (Additional file 1: Table S2). No disease mutations with ‘confirmed’ status in the current MITOMAP database [4] were found in any individuals.

The lowest heteroplasmy level of mtDNA variants was 10% for several non-coding variants. The levels of heteroplasmy of some mutations (m.3206C > T, m.4824A > G, m.8473T > C, m.16179CA > C, and m.16183A > C) varied from approximately 10% to 100% (homoplasmy) and their allele frequency in the studied population was 11%. The m.16189T > C polymorphism causing a lower mtDNA copy number was present in 29/118 (24.58%) samples as homoplasmy, and in one healthy individual at a heteroplasmy level of 54.6% (Additional file 1: Table S3).

### Classification of mitochondrial haplogroups in the Korean population

**Fig. 3:**
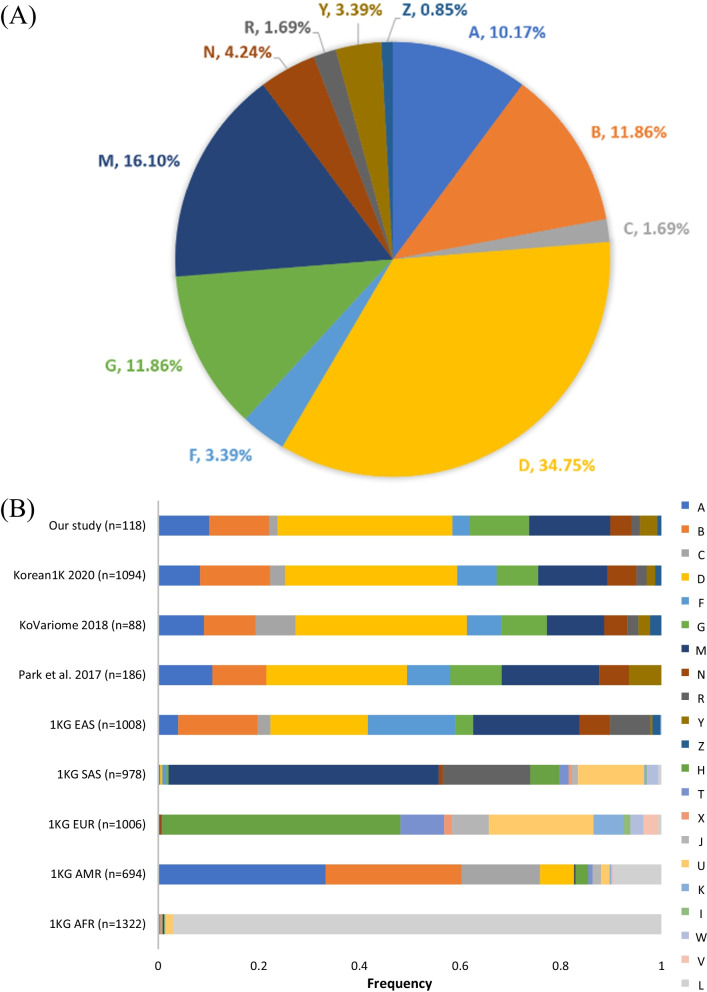
The haplogroup of mitochondrial genomes for Korean population. **A** Distribution of haplogroups in the Korean population in the present study. **B** Stacked bar plots of the frequencies of haplogroups in the Korean population and five super-populations from the 1000 Genomes Project (1 KG)

We examined all haplogroups previously reported in the Korean population in Korean Genome Project (Korea 1K) (*n* = 1094) [23], and listed in the Korean National Standard Reference Variome Database of whole genomes (KoVariome) [43]. Eleven variants (m.73A > G, m.263A > G, m.750A > G, m.1438A > G, m.2706A > G, m.4769A > G, m.7028C  > T, m.8860A > G, m.11719G > A, m.14766C > T, and m.15326A > G) with an overall frequency ≥ 50% that are widespread across all lineages (L, M, and N) [44] were predominant in our study. Among these, five mtDNA variants present at ≥ 80% in lineages L, M, or N were found in all individuals. The rare mutation m.73A > C was observed in one individual in our population. The most dominant haplogroup for the Korean population was found to be D (34.74%), followed by M (16.1%). These haplogroups are prevalent in Asian populations [45] and were diverse in our study subgroups (Fig. 3A, Table 1). The D2 sub-haplogroup, defined by mutation m.16271T > C [46] and possibly equating to D4e1b [47], was observed in one subject. Consequently, 87.8% of haplogroup D belonged to the D4 subgroup, which had the highest frequency in our Korean population; the D5 subgroup was observed in all remaining subjects. These results were consistent with the data of Korea 1K, which includes sequence data from 1094 whole genomes.

**Table 1:** The sub-haplogroups observed in our study (n = 118) based on entire mitochondrial genome variants by using PhyloTree 17 nomenclature in Haplogrep2 analysis

Haplogroup	Sub-haplogroup	Number of subjects (n = 118)
A	A + 152 + 16,362, A1, A11, A5a, A5a1a, A5b1a, A5b1b, A6	12 (10.17%)
B	B4a1b1a, B4a1c1a1, B4a4, B4b1a2, B4b1a2a, B4c1a1a, B4c1a1b, B4d3a1, B4f1, B5a2a1 + 16,129, B5b, B5b1, B5b2a2	14 (11.86%)
C	C4a1a, C7a1c,	2 (1.69%)
D	D2, D4, D4 + 195, D4a, D4a1, D4a1c, D4a1h, D4a3b, D4a3b1, D4b2, D4b2a2a1, D4b2b, D4b2b1, D4c1a, D4c2c, D4e1a, D4e2, D4f1, D4g1, D4g1a, D4g1b, D4g1c, D4h1a, D4h1c1, D4i, D4n, D5a, D5a2a1 + 16,172, D5b, D5b1, D5b1b	41 (34.75%)
F	F1a1, F2, F2f, F2i,	4 (3.39%)
G	G1a1, G1a1a, G1a1a1, G2a + 152, G2a1, G2a1 + 16,189 + 16,194, G2a1b, G2a1e, G2a5, G2b2	14 (11.86%)
M	M10, M10a1a1b, M10a1b, M11b1a, M7a1a, M7a1a1, M7a1a5, M7b1a1a1, M7c1a2a1, M7c1a3, M7c1a5, M7c1b, M8a3a, M8a3a1, M9a1a1, M9a1a1a	19 (16.1%)
N	N9a2, N9a2a, N9a2c, N9a2d	5 (4.24%)
R	R + 16,189, R11a	2 (1.69%)
Y	Y1	4 (3.39%)
Z	Z4a	1 (0.85%)

The distributions of the haplogroups in the Korean population were obtained from Korea 1K [23], KoVariome [43] and five super-populations in the 1000 Genomes Project (1 KG) [33] and then compared to our data. Our study showed the concordance of the haplogroups of the Korean and East Asian populations (1 KG-EAS) (Fig. 3B). Other super-populations had distinct mtDNA haplogroups, as described elsewhere [19]. Haplogroups M, A, H and L were most prevalent in South Asian (1 KG-SAS), American (1 KG-AMR), European (1 KG-EUR) and African (1 KG-AFR) populations, respectively.

### Annotation of mtDNA variants and drug-induced toxicity

The D-loop is 1122 bp in length, and consists of two hypervariable regions (HVI at nucleotides 16,024–16,383 and HVII at nucleotides 57–372) and a tandem repeat of poly(C) at nucleotides 303–315 [48]. In all subjects, the mtDNA D-loop region had 74 polymorphisms. All 118 samples had the polymorphism m.263A > G in HVII and one subject carried m.73A > C, which was reported to be rare in Asian p opulations (0.01% in healthy individuals and 0.09% in patients). In 30/118 (25.42%) of the samples in the present study, a thymine to cytosine (T > C) transition was found at position 16,189, producing an uninterrupted homopolymeric tract at mtDNA segment 16,180–16,195 in HVI. In total, 3 of the 118 samples (2.5%) had 12 uninterrupted cytosine residues, with the heteroplasmy level of m.16189T > C varying from 54.6% to 99.9%. Meanwhile, 2 of the 118 (1.7%) samples had length heteroplasmy in the mitochondrial HVII segment at positions 303–315 due to the insertion of cytosine at position 309, and one subject showed replication slippage in the case of a T > C transition at position 310.

We observed a total of 40 mitochondrial variants in mitochondrially encoded 12S rRNA (*MT-RNR1*) and mitochondrially encoded 16S rRNA (*MT-RNR2*) genes. Several mtDNA mutations associated with antibiotic-induced toxicity were detected in these two genes (Table 2). The m.1555A > G and m.1494C > T mutations are well known to be associated with ototoxicity in patients treated with aminoglycoside antibiotics, but were not observed in our population. We may not have been able to detect these rare variants, reported to be found at a rate of 2 per 2922 individuals (0.07%) for m.1555A > G in the Korean population [49], due to the small sample size. Other aminoglycoside-induced hearing loss variant, m.1189T > C, was observed in 1.69% of all samples; the rate was previously reported as 0.34% for the Korean population [50, 51]. Variant at position 961 was identified at a frequency of 5.93%; these are more likely to be found in Korean than Asian populations (1–3% in the 1 KG). The polymorphism of m.2706A > G transition, a variation previously suggested to lead to a pred isposition to linezolid-associated lactic acidosis [52], was dominant in all subjects. The m.3010G > A allele, which is associated with linezolid-induced mitochondrial toxicity, was more common in the Korean population (30.51%), consistent with previous studies [45, 53]. Lastly, we observed a high frequency of m.10398A > G variant that was reported to influence metabolic ART effects [15] at homoplasmic level in our study.

**Table 2:** Frequencies of mitochondrial variants in association with drug-induced toxicity in this study (n = 118)

Gene	Variant	rsID	Drug	Clinically relevant	Frequency in our study (%)
*MT-RNR1*	m.663A > G	rs56489998	Aminoglycoside	Ototoxicity [51]	10.17
	m.961T > C	rs3888511	Aminoglycoside	Ototoxicity [51]	5.93
	m.961T > C	rs3888511	Linezolid	Mitochondrial toxicity [54]	5.93
	m.1095T > C	rs267606618	Aminoglycoside	Ototoxicity [51]	0.85
	m.1189T > C	rs28358571	Aminoglycoside	Ototoxicity [51]	1.69
*MT-RNR2*	m.2706A > G	rs2854128	Linezolid	Lactic acidosis, Mitochondrial toxicity [54]	99.15
	m.3010G > A	rs3928306	Linezolid	Mitochondrial toxicity [54]	30.51
*MT-ND3*	m.10398A > G	NA	ART	Metabolic/ cardiovascular complications [15]	70.34

^*^MT-RNR1: Mitochondrially encoded 12S ribosomal RNA; *MT-RNR2*: Mitochondrially encoded 16S ribosomal RNA; *MT-ND3*: Mitochondrially encoded NADH: Ubiquinone Oxidoreductase Core Subunit 3

## Discussion

The D-loop has a regulatory role in mtDNA transcription and replication [55]. Mutations in the D-loop region may significantly affect mtDNA copy number and the gene expression of mitochondrial genomes, thereby disrupting the function of mitochondria, oxidative phosphorylation and ATP production. Here, we found the largest number of polymorphisms in the D-loop region in all subjects. Notably, 25.42% of the samples harboured the mutation m.16189T > C, which is very close to the mtDNA origin of replication and is significantly associated with various non-communicable diseases [56–59]. This could be explained by the lower binding affinity of proteins to the regi on with the 16189C variant [56]. A negative correlation between continuous cytosines at the mtDNA segment 16,180–16,195 in HVI and mtDNA copy number was reported previously in human peripheral blood cells [60]. The reduction of mitochondrial copy number might lead to the mitochondrial dysfunction that is potentially a mechanism of drug-induced toxicity [61]. It suggested that m.16180T > C variant might increase the risk of developing adverse drug reaction. Thus, the impact of this variant should be considered in further investigation.

The non-synonymous m.10398A > G polymorphism (overall frequency = 70.34%) was reported to be associated with long-term ART-induced toxicity. Notably, most of the variants mentioned above showed homoplasmy (> 98.4% of all mtDNA sequences analysed [62]). The phenotypic expression of pathogenic and heteroplasmic mtDNA mutations could be modulated by homoplasmic mtDNA variants [15]. This suggests that determination of these variants prior to treatment could be useful to identify tuberculosis and HIV patients at risk of drug-induced toxicity.

The frequency of length heteroplasmy in the homopolymeric C-stretch regions was as low as ~ 4% in our population, and we detected only one individual with possible length heteroplasmy at position 303–315. Compared to point heteroplasmy, length heteroplasmy is more common and less population-specific [63]. The presence of a tandem poly(C) repeat in the D-loop region of nucleotides 303–315 and 16,182–16,189, as a result of polymerase misincorporation or slippage during mtDNA replication [64], reduced the mtDNA copy number. These length heteroplasmies were reportedly associated with a more than twofold increase in cancer risk [65]. In addition, these length variants have been suggested to be associated with several common diseases, including diabetes mellitus and dilated cardiomyopathy [66], and are therefore of clinical importance.

The human mitochondrial genome consists of 13 protein-coding genes, 22 tRNAs and 2 rRNAs. The proteins encoded by the 13 mitochondrial-coding genes are constituents of the enzyme complexes of the oxidative phosphorylation system, enabling mitochondria to act as the cellular powerhouse. They are distributed among various respiratory chain components, in a small part of complex I and throughout complexes III–V (ATP synthase) of the respiratory chain [67].

Mitochondrial rRNA more closely resembles bacterial than human rRNA. However, genetic polymorphisms in these genes can result in greater similarity between mitochondrial and bacterial rRNA, thereby facilitating the binding of anti-microbials (most notably aminoglycosides). In cases of tuberculosis infection treated via multi-antibiotic administration, the associations of mtDNA instability and variation with drug-induced toxicity should be taken into consideration. The polymorphisms in the *MT-RNR1* and *MT-RNR2* genes were associated with increased mitochondrial and clinical adverse effects, most commonly ototoxicity and peripheral neuropathy [54, 68]. The mutation of mitochondrial 12S rRNA gene is associated with both aminoglycoside-induced deafness and non-syndromic hearing loss. This study detected higher allele frequency in some variants (m.663A > G, m.961T > C, m.3010G > A) compared to previous studies [15, 46]. This suggested that these variants should be considered carefully in studies of the Korean population. It has recently been reported that mtDNA polymorphisms may affect respiratory chain function and first-line anti-tuberculosis drug-induced liver injury. However, the underlying mechanisms have yet to be elucidated [69, 70].

The rare mutations observed in our clinical samples may have highly disruptive effects on mitochondrial ribosomes; based on previous study, potential disruption is classified as ‘proven’ or ‘not enough evidence’ (NEE) [71, 72]. Among 52 and 145 mutations in the *MT-RNR1* and *MT-RNR2* genes, respectively, only three (m.1494C > T, m.1555A > G and m.1843T > C) were ‘proven’ and some of those variants were interpreted in clinical practice for *MT-RNR1* gene [51]. Most of the remaining variants were classified as NEE, so it is not yet clear whether changes in mitochondrial ribosomes affect the binding sites of antibiotic drugs to reduce drug toxicity. Other mtDNA-encoded ribosome components were suggested to have potential benefits in terms of drug susceptibility and should therefore be investigated further in clinical studies. In the present study, only m.2150T > A, classified as NEE, was observed (at a rate of 4.24%; 5/118 samples) as a homoplasmic mutant.

In addition, adverse drug reactions were reported in patients inheriting pathogenic mtDNA mutations. Antibiotic drugs were reported to be associated with optic neuropathy in Leber’s hereditary optic neuropathy (LHON) carriers. In patients carrying the m.11778G > A (*MT-ND4*) mutation, erythromycin has the potential to catalyse a bioenergetic crisis at onset of LHON [73], while ethambutol was suggested to have a synergistic and deleterious effect on tissue specificity, as reviewed elsewhere [74]. Therefore, the use of erythromycin and, by extension, other macrolides, should be avoided in patients with pre-existing pathogenic mtDNA mutations.

The polymorphism of mitochondrial genome has been shown to be associated with v arious pathophysiological conditions, and could play a role in various physiological and pathological characteristics [3]. The high variability in human mitochondria has been investigated for common diseases [2]. At the time of study, ninety-five mitochondrial markers have been confirmed to be pathogenic [4]. Pathogenic mtDNA mutations were commonly found in a number of major haplogroups in the general population, indicating that many healthy individuals carry these potentially harmful mtDNA mutations. Therefore, mtDNA mutations could serve as biomarkers facilitating early detection and prediction of the prognosis of disease. However, our study identified no pathogenic variants, possibly due to its small sample size.

In European populations, it has been reported that specific mtDNA haplogroups were associated with ART-induced peripheral neuropathy and metabolic disorders [10–15]. In addition, many studies have demonstrated relationships between haplogroups and cancer risk. However, at the time of our study, there had been no investigations of the relationships between haplogroups and drug-induced toxicity for Korean population. Haplogroups vary widely across ethnic groups, even between South and East Asians. Therefore, the characteristics of haplogroups in specific populations should be considered when exploring the susceptibility of these populations to diseases and drug-induced toxicity.

In this study, we aimed to characterizing the mitochondrial genome profile for Korean population and observed only few known-mutations related to ADRs that could be the explanation for the absence of side effects in 68 subjects who were treated with the first-line antituberculosis drug. However, the characterized variants might contribute to ADRs related to other drugs. For future direction, we choose the subjects to further explore the association of mitochondrial variants with ADRs. In addition to mitochondrial polymorphism, many other factors including nucleus genes, comorbidity, comedication etc. could induce mitochondrial dysfunction that leads to ADRs. Consideration of all these factors will allow us to explore the insight of the association between mtDNA variants and drug-induced toxicity in future study.

## Conclusions

Drug-induced toxicity via mitochondrial dysfunction has been studied extensively. However, the contributions of mitochondrial variants to drug-induced toxicity remain unclear. The present study identified distinct haplotypes in a Korean population, consistent with the results reported previously by Korea 1K. Despite the relatively small sample size of our study, we investigated annotation between mtDNA characteristics and risk factors for certain diseases and drug-induced toxicity, through NGS analysis. Further studies including larger cohorts are needed to examine the associations of mtDNA with adverse drug reactions in the Korean population. For this issue, we are going to extend the investigation in association with antituberculosis drug-induced toxicity such as drug-induced liver injury and peripheral neuropathy.

## Supplementary Information

**Additional file 1.** Supplementary material.

## Data Availability

The datasets generated during the current study are available in the PRJNA774877 repository at this link: https://www.ncbi.nlm.nih.gov/sra/?term=PRJNA774877.
