# Supplementary material for: The frequency of the known mitochondrial variants associated with drug-induced toxicity in a Korean population
Source: BMC Med Genomics. 2022 Jan 3;15:3. doi: 10.1186/s12920-021-01153-0 (PMC8722126; doi:10.1186/s12920-021-01153-0)
Supplement: Supplementary file 1 — Additional file 1. Supplementary material. [file 12920_2021_1153_MOESM1_ESM.docx]

**
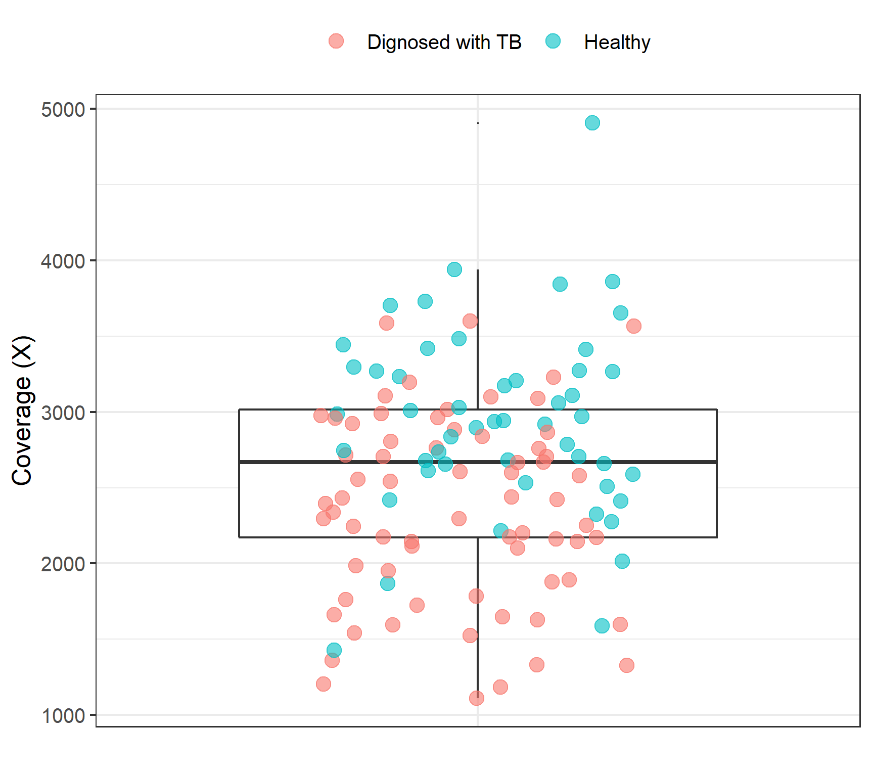
**

**Figure S1**: The mean coverage of mitochondrial genome sequence for each sample in our study (n = 118). TB: tuberculosis.

**Table S1**: Characteristic of studied subjects in this study (n = 118).

| **Characteristic** | **All subjects**  **(n = 118)** | **Healthy subjects**  **(n= 50)** | **Subjects diagnosed with tuberculosis (n = 68)** |
| --- | --- | --- | --- |
| Male (%) | 65 (55%) | 21 (42%) | 44 (64.7%) |
| Age (year)^*^ | 37.1 (19-65) | 29.8 (21 - 57) | 42.4 (19 - 65) |
| BMI (kg.m-2) ^*^ | 22.1 (15.1 – 34.1) | 22.2 (16.9 - 32) | 22 (15.1 – 34.1) |
| Smoking status |  |  |  |
| Non-smoking | 88 (74.6%) | 42 (84%) | 46 (67.6%) |
| Smoking | 30 (25.4%) | 8 (16%) | 22 (32.3%) |
| TB_diagnosis |  |  |  |
| Lung |  |  | 57 (83.8%) |
| Lymph nodes |  |  | 4 (5.9%) |
| Pleura |  |  | 4 (5.9%) |
| Abdomen |  |  | 1 (1.5%) |
| Bronchus |  |  | 1 (1.5%) |
| Other |  |  | 1 (1.5%) |
| TB_treatment |  |  |  |
| Initial treatment |  |  | 56 (82%) |
| Re-treatment |  |  | 12 (18%) |

** Data was shown as median (min – max)*

**Table S2**: Total mtDNA variants detected in each locus of mitochondrial genome in our study (n=118)

| **Category** | **Locus** | **Our study (n = 118)** |
| --- | --- | --- |
| D-loop | D-loop | 74 |
| Complex V: ATP synthase | *MT-ATP6^†^* | 28 |
|  | *MT-ATP6-8^†^* | 3 |
|  | *MT-ATP8^†^* | 9 |
| Complex IV: Cytochrome c oxidase | *MT-COX1^†^* | 38 |
|  | *MT-COX2^†^* | 31 |
|  | *MT-COX3^†^* | 30 |
| Complex III: Coenzyme Q - cytochrome c reductase / Cytochrome b | *MT-CYTB^†^* | 58 |
| Complex I: NADH dehydrogenase | *MT-ND1^†^* | 36 |
|  | *MT-ND2^†^* | 32 |
|  | *MT-ND3^†^* | 11 |
|  | *MT-ND4^†^* | 43 |
|  | *MT-ND4L^†^* | 9 |
|  | *MT-ND5^†^* | 66 |
|  | *MT-ND6^†^* | 20 |
| Mitochondrial rRNA coding genes | *MT-RNR1^†^* | 31 |
|  | *MT-RNR2^†^* | 21 |
| Mitochondrial tRNA coding genes | *MT-TRNA^†^* | 4 |
|  | *MT-TRNC^†^* | 1 |
|  | *MT-TRNE^†^* | 3 |
|  | *MT-TRNG^†^* | 2 |
|  | *MT-TRNI^†^* | 1 |
|  | *MT-TRNM^†^* | 1 |
|  | *MT-TRNN^†^* | 2 |
|  | *MT-TRNP^†^* | 113 |
|  | *MT-TRNQ^†^* | 3 |
|  | *MT-TRNR^†^* | 2 |
|  | *MT-TRNS2^†^* | 1 |
|  | *MT-TRNT^†^* | 6 |
|  | *MT-TRNY^†^* | 2 |
| **Total variants** |  | 681 |

*^†^ MT-ATP6, MT-ATP8*: mitochondrially encoded ATP synthase membrane subunit 6, 8 genes; *MT-COX1, MT-COX2, MT-COX3*: mitochondrially encoded cytochrome C oxidase I, II, III genes, respectively; *MT-CYTB*: mitochondrially encoded cytochrome B gene; *MT-ND1, MT-ND2, MT-ND3, MT-ND4, MT-ND4L, MT-ND5, MT-ND6*: mitochondrially encoded NADH: ubiquinone oxidoreductase core subunit 1, 2, 3, 4, 4L, 5, 6 genes, respectively; *MT-RNR1, MT-RNR2*: mitochondrially encoded 12S, 16S ribosomal RNA genes, respectively; *MT-TRNA, MT-TRNC, MT-TRNE, MT-TRNG, MT-TRNI, MT-TRNM, MT-TRNN, MT-TRNP, MT-TRNQ, MT-TRNR, MT-TRNS2, MT-TRNT, MT-TRNY*: mitochondrial encoded tRNA genes.

**Table S3**: Heteroplasmy level of mtDNA variants with allele frequency (AF) > 5% in our study (n=118). The heteroplasmy was presented as min – max value of heteroplasmy level.

| **POS**^†^ | **Locus** | **REF**^‡^ | **ALT**^⁋^ | **TYPE**^⁎^ | **AF**^⁑^ **(%)** | **Heteroplasmy** |
| --- | --- | --- | --- | --- | --- | --- |
| 73 | D-loop | A | G | SNP | 99.15 | 0.987 - 0.999 |
| 146 | D-loop | T | C | SNP | 16.95 | 0.111 - 0.999 |
| 150 | D-loop | C | T | SNP | 16.95 | 0.797 - 0.999 |
| 152 | D-loop | T | C | SNP | 24.58 | 0.651 - 1 |
| 195 | D-loop | T | C | SNP | 6.78 | 0.993 - 0.999 |
| 199 | D-loop | T | C | SNP | 8.47 | 0.994 - 1 |
| 207 | D-loop | G | A | SNP | 5.08 | 0.965 - 0.999 |
| 235 | D-loop | A | G | SNP | 10.17 | 0.993 - 0.999 |
| 247 | D-loop | GA | G | INDEL | 5.93 | 0.997 - 0.999 |
| 263 | D-loop | A | G | SNP | 100.00 | 0.994 - 0.999 |
| 302 | D-loop | A | AC | INDEL | 55.08 | 0.104 - 0.896 |
| 302 | D-loop | A | ACC | INDEL | 33.05 | 0.102 - 0.845 |
| 302 | D-loop | A | ACCC | INDEL | 9.32 | 0.115 - 0.346 |
| 310 | D-loop | T | TC | INDEL | 83.05 | 0.932 - 0.998 |
| 489 | D-loop | T | C | SNP | 65.25 | 0.981 - 0.999 |
| 513 | D-loop | GCA | G | INDEL | 32.20 | 0.991 - 0.999 |
| 567 | D-loop | A | ACCCC | INDEL | 6.78 | 0.155 - 0.412 |
| 567 | D-loop | A | ACCCCC | INDEL | 6.78 | 0.14 - 0.468 |
| 567 | D-loop | A | ACCC | INDEL | 5.08 | 0.116 - 0.314 |
| 663 | MT-RNR1 | A | G | SNP | 10.17 | 0.993 - 0.999 |
| 709 | MT-RNR1 | G | A | SNP | 26.27 | 0.99 - 0.999 |
| 750 | MT-RNR1 | A | G | SNP | 100.00 | 0.991 - 1 |
| 827 | MT-RNR1 | A | G | SNP | 5.08 | 0.996 - 1 |
| 955 | MT-RNR1 | A | ACCCC | INDEL | 5.08 | 0.109 - 0.452 |
| 955 | MT-RNR1 | A | AC | INDEL | 5.93 | 0.261 - 0.341 |
| 955 | MT-RNR1 | A | ACC | INDEL | 5.93 | 0.151 - 0.255 |
| 961 | MT-RNR1 | T | C | SNP | 5.93 | 0.979 - 0.995 |
| 1438 | MT-RNR1 | A | G | SNP | 99.15 | 0.992 - 1 |
| 1598 | MT-RNR1 | G | A | SNP | 5.08 | 0.995 - 0.999 |
| 1736 | MT-RNR2 | A | G | SNP | 9.32 | 0.993 - 0.999 |
| 2706 | MT-RNR2 | A | G | SNP | 99.15 | 0.988 - 0.999 |
| 3010 | MT-RNR2 | G | A | SNP | 30.51 | 0.991 - 0.999 |
| 3206 | MT-RNR2 | C | T | SNP | 11.86 | 0.102 - 0.998 |
| 3834 | MT-ND1 | G | A | SNP | 5.08 | 0.813 - 0.999 |
| 4071 | MT-ND1 | C | T | SNP | 5.93 | 0.996 - 0.999 |
| 4248 | MT-ND1 | T | C | SNP | 10.17 | 0.99 - 0.997 |
| 4769 | MT-ND2 | A | G | SNP | 97.46 | 0.991 - 0.999 |
| 4824 | MT-ND2 | A | G | SNP | 11.02 | 0.284 - 0.999 |
| 4833 | MT-ND2 | A | G | SNP | 11.86 | 0.984 - 0.999 |
| 4883 | MT-ND2 | C | T | SNP | 34.75 | 0.993 - 0.999 |
| 5108 | MT-ND2 | T | C | SNP | 11.86 | 0.982 - 1 |
| 5178 | MT-ND2 | C | A | SNP | 34.75 | 0.989 - 1 |
| 5231 | MT-ND2 | G | A | SNP | 5.08 | 0.995 - 0.998 |
| 5301 | MT-ND2 | A | G | SNP | 5.08 | 0.99 - 0.998 |
| 5417 | MT-ND2 | G | A | SNP | 7.63 | 0.994 - 0.999 |
| 5601 | MT-TRNA | C | T | SNP | 7.63 | 0.972 - 0.998 |
| 5894 | MT-TRNY | A | AC | INDEL | 5.08 | 0.868 - 0.989 |
| 6455 | MT-COX1 | C | T | SNP | 8.47 | 0.989 - 1 |
| 7028 | MT-COX1 | C | T | SNP | 100.00 | 0.991 - 0.999 |
| 7600 | MT-COX2 | G | A | SNP | 6.78 | 0.982 - 0.999 |
| 8270 | MT-COX2 | CACCCCCTCT | C | INDEL | 12.71 | 0.984 - 0.999 |
| 8414 | MT-ATP8 | C | T | SNP | 30.51 | 0.981 - 0.999 |
| 8473 | MT-ATP8 | T | C | SNP | 11.86 | 0.101 - 0.998 |
| 8563 | MT-ATP6-8 | A | G | SNP | 5.93 | 0.996 - 0.999 |
| 8584 | MT-ATP6 | G | A | SNP | 9.32 | 0.991 - 0.998 |
| 8701 | MT-ATP6 | A | G | SNP | 61.02 | 0.977 - 0.999 |
| 8794 | MT-ATP6 | C | T | SNP | 10.17 | 0.993 - 0.998 |
| 8860 | MT-ATP6 | A | G | SNP | 27.97 | 0.994 - 0.999 |
| 9377 | MT-COX3 | A | G | SNP | 6.78 | 0.987 - 0.999 |
| 9540 | MT-COX3 | T | C | SNP | 65.25 | 0.984 - 1 |
| 9575 | MT-COX3 | G | A | SNP | 6.78 | 0.981 - 0.998 |
| 9824 | MT-COX3 | T | C | SNP | 8.47 | 0.996 - 1 |
| 9950 | MT-COX3 | T | C | SNP | 5.08 | 0.993 - 0.999 |
| 10310 | MT-ND3 | G | A | SNP | 5.08 | 0.994 - 0.999 |
| 10398 | MT-ND3 | A | G | SNP | 70.34 | 0.983 - 1 |
| 10400 | MT-ND3 | C | T | SNP | 65.25 | 0.983 - 1 |
| 10873 | MT-ND4 | T | C | SNP | 64.41 | 0.901 - 0.999 |
| 11536 | MT-ND4 | C | T | SNP | 5.93 | 0.995 - 0.997 |
| 11719 | MT-ND4 | G | A | SNP | 100.00 | 0.993 - 0.999 |
| 11914 | MT-ND4 | G | A | SNP | 7.63 | 0.988 - 0.998 |
| 12358 | MT-ND5 | A | G | SNP | 5.08 | 0.994 - 0.999 |
| 12372 | MT-ND5 | G | A | SNP | 5.08 | 0.994 - 0.999 |
| 12705 | MT-ND5 | C | T | SNP | 83.05 | 0.929 - 0.999 |
| 13563 | MT-ND5 | A | G | SNP | 7.63 | 0.992 - 0.999 |
| 14200 | MT-ND6 | T | C | SNP | 5.93 | 0.995 - 0.999 |
| 14569 | MT-ND6 | G | A | SNP | 11.86 | 0.993 - 0.999 |
| 14668 | MT-ND6 | C | T | SNP | 30.51 | 0.935 - 0.999 |
| 14766 | MT-CYTB | C | T | SNP | 100.00 | 0.989 - 1 |
| 14783 | MT-CYTB | T | C | SNP | 65.25 | 0.93 - 1 |
| 14979 | MT-CYTB | T | C | SNP | 11.02 | 0.932 - 0.999 |
| 15043 | MT-CYTB | G | A | SNP | 66.10 | 0.932 - 0.998 |
| 15301 | MT-CYTB | G | A | SNP | 65.25 | 0.932 - 1 |
| 15326 | MT-CYTB | A | G | SNP | 99.15 | 0.996 - 1 |
| 16093 | MT-TRNP | T | C | SNP | 8.47 | 0.956 - 0.994 |
| 16126 | MT-TRNP | T | C | SNP | 5.93 | 0.333 - 0.999 |
| 16129 | MT-TRNP | G | A | SNP | 20.34 | 0.975 - 0.999 |
| 16172 | MT-TRNP | T | C | SNP | 6.78 | 0.989 - 0.999 |
| 16179 | MT-TRNP | CA | C | INDEL | 13.56 | 0.141 - 0.978 |
| 16179 | MT-TRNP | CAA | C | INDEL | 10.17 | 0.183 - 0.683 |
| 16182 | MT-TRNP | AA | CC | MNP | 9.32 | 0.129 - 0.494 |
| 16183 | MT-TRNP | A | C | SNP | 14.41 | 0.152 - 0.797 |
| 16183 | MT-TRNP | A | ACCC | INDEL | 6.78 | 0.232 - 0.362 |
| 16187 | MT-TRNP | C | T | SNP | 5.08 | 0.993 - 0.999 |
| 16188 | MT-TRNP | CT | C | INDEL | 7.63 | 0.135 - 0.974 |
| 16189 | MT-TRNP | T | C | SNP | 25.42 | 0.546 - 0.999 |
| 16217 | MT-TRNP | T | C | SNP | 7.63 | 0.989 - 0.999 |
| 16223 | MT-TRNP | C | T | SNP | 78.81 | 0.974 - 0.999 |
| 16234 | MT-TRNP | C | T | SNP | 5.93 | 0.993 - 0.999 |
| 16261 | MT-TRNP | C | T | SNP | 7.63 | 0.993 - 0.997 |
| 16278 | MT-TRNP | C | T | SNP | 13.56 | 0.99 - 0.999 |
| 16290 | MT-TRNP | C | T | SNP | 10.17 | 0.993 - 0.999 |
| 16311 | MT-TRNP | T | C | SNP | 12.71 | 0.96 - 0.999 |
| 16319 | MT-TRNP | G | A | SNP | 15.25 | 0.99 - 0.999 |
| 16325 | MT-TRNP | T | C | SNP | 5.08 | 0.993 - 0.999 |
| 16362 | MT-TRNP | T | C | SNP | 54.24 | 0.644 - 1 |
| 16519 | MT-TRNP | T | C | SNP | 48.31 | 0.974 - 0.999 |

POS^†^: position of SNP in mtDNA genome;

REF^‡^: reference sequence from Revised Cambridge Reference Sequence of the Human Mitochondrial DNA (NC_012920.1);

ALT^⁋^: mitochondrial variants that were observed in our study;

TYPE^⁎^: the type of variants (SNP: single nucleotide polymorphism, INDEL: insertion and/or deletion mutation);

AF^⁑^: allele frequency that was detected in our study (%).
